# Phospholipid Metabolism Is Associated with Time to HIV Rebound upon Treatment Interruption

**DOI:** 10.1128/mBio.03444-20

**Published:** 2021-02-23

**Authors:** Leila B. Giron, Emmanouil Papasavvas, Xiangfan Yin, Aaron R. Goldman, Hsin-Yao Tang, Clovis S. Palmer, Alan L. Landay, Jonathan Z. Li, John R. Koethe, Karam Mounzer, Jay R. Kostman, Qin Liu, Luis J. Montaner, Mohamed Abdel-Mohsen

**Affiliations:** a The Wistar Institute, Philadelphia, Pennsylvania, USA; b The Burnet Institute, Melbourne, Victoria, Australia; c Department of Infectious Diseases, Monash University, Melbourne, Victoria, Australia; d Rush University, Chicago, Illinois, USA; e Department of Medicine, Brigham and Women’s Hospital, Harvard Medical School, Boston, Massachusetts, USA; f Vanderbilt University, Nashville, Tennessee, USA; g Philadelphia FIGHT, Philadelphia, Pennsylvania, USA; University of Pittsburgh School of Medicine

**Keywords:** HIV, HIV persistence, lipids, lysophospholipid, viral rebound, lysophosphatidylcholine, phospholipid, TMAO, choline

## Abstract

Lipids are biologically active molecules involved in a variety of cellular processes and immunological functions, including inflammation. It was recently shown that phospholipids and their derivatives, lysophospholipids, can reactivate latent (dormant) tumor cells, causing cancer recurrence. However, the potential link between lipids and HIV latency, persistence, and viral rebound after cessation of antiretroviral therapy (ART) has never been investigated. We explored the links between plasma lipids and the burden of HIV during ART. We profiled the circulating lipidome from plasma samples from 24 chronically HIV-infected individuals on suppressive ART who subsequently underwent an analytic treatment interruption (ATI) without concurrent immunotherapies. The pre-ATI viral burden was estimated as time-to-viral-rebound and viral load set points post-ATI. We found that higher pre-ATI levels of lysophospholipids, including the proinflammatory lysophosphatidylcholine, were associated with faster time-to-viral-rebound and higher viral set points upon ART cessation. Furthermore, higher pre-ATI levels of the proinflammatory by-product of intestinal lysophosphatidylcholine metabolism, trimethylamine-*N*-oxide (TMAO), were also linked to faster viral rebound post-ART. Finally, pre-ATI levels of several phosphatidylcholine species (lysophosphatidylcholine precursors) correlated strongly with higher pre-ATI levels of HIV DNA in peripheral CD4^+^ T cells. Our proof-of-concept data point to phospholipids and lysophospholipids as plausible proinflammatory contributors to HIV persistence and rapid post-ART HIV rebound. The potential interplay between phospholipid metabolism and both the establishment and maintenance of HIV latent reservoirs during and after ART warrants further investigation.

## OBSERVATION

A comprehensive understanding of the host factors modulating HIV persistence is imperative for developing effective strategies to eradicate the latent HIV reservoir, which persists despite antiretroviral therapy (ART) and causes viral rebound upon ART discontinuation (1). Lipids are biologically active molecules involved in a broad range of cellular processes and immunological functions, including inflammation (2, 3). It was recently shown that phospholipids and their derivatives, lysophospholipids, can reactivate latent (dormant) tumor cells, causing cancer recurrence (4). While the interplay between lipids and both HIV and ART has been studied in the context of the development of inflammation-associated comorbidities, particularly subclinical atherosclerosis (5–9), the potential impact of lipids on HIV latency, persistence, and post-ART rebound has never been investigated.

There is currently no standard method to measure the total body burden of the replication-competent HIV reservoir (1, 10). However, a possible way to estimate both the overall size of the HIV reservoir and the degree of viral control is by assessing time-to-viral-rebound and/or viral load set points upon cessation of ART. In this study, we profiled the circulating lipidome from plasma samples from 24 chronically HIV-infected individuals on suppressive ART who subsequently underwent an analytic treatment interruption (ATI) (11, 12). All 24 individuals underwent ATI without concurrent immunomodulatory agents that might confound our analysis. Lipidomic analysis was performed using liquid chromatography-mass spectrometry (LC-MS), as described previously (13), on plasma samples collected immediately before ATI. Both time-to-viral-rebound and viral load set points were measured during ATI. This cohort had a wide distribution of viral rebound times (14 to 119 days; median = 28) and viral load set points (median = 13,675 copies/ml; see Table S1 in the supplemental material). Using these data, we investigated whether there is a link between pre-ATI lipid profiles and the body burden of HIV during ART (estimated as post-ATI time-to-viral-rebound and viral load set points).

10.1128/mBio.03444-20.1 TABLE S1 Clinical and demographic data of the study cohort. Download Table S1, PDF file, 0.04 MB. Copyright © 2021 Giron et al. 2021 Giron et al. https://creativecommons.org/licenses/by/4.0/ This content is distributed under the terms of the Creative Commons Attribution 4.0 International license.

### Levels of plasma lysophospholipids measured pre-ATI associate with time to viral rebound post-ATI.

**FIG 1:**
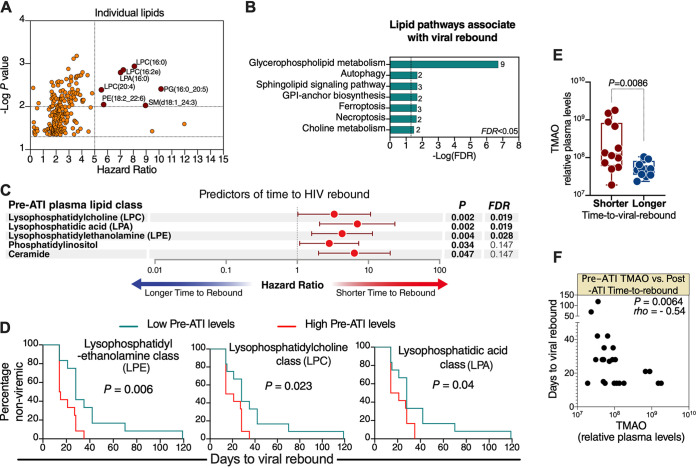
Higher pre-ATI lysophospholipid metabolism and its bioactive by-products associate with faster post-ATI time to viral rebound. (A) Lipids whose pre-ATI levels associate with post-ATI time to viral rebound, as determined by the Cox proportional-hazards model. Lipids with *P* < 0.01 and hazard ratio (HR) > 5 are shown in red and labeled. (B) Lipid pathway analysis of plasma lipids whose pre-ATI levels associated with time to viral rebound with *P* < 0.05 using LIPEA (Lipid Pathway Enrichment Analysis; https://lipea.biotec.tu-dresden.de/home). The graph shows all implicated pathways with FDR < 0.05. Numbers beside each pathway represent the number of dysregulated lipids within the particular pathway. GPI, glycosylphosphatidylinositol. (C) Lipid classes whose pre-ATI levels associate with post-ATI time to viral rebound, as determined by the Cox proportional-hazards model. FDR was calculated using the Benjamini-Hochberg approach. (D) Confirmatory analysis of the three lysophospholipid classes using the Mantel-Cox test. Low pre-ATI levels are levels lower than the group median; high pre-ATI levels are levels higher than the group median. (E) Participants were separated into shorter or longer time-to-viral-rebound groups by the median of days to viral rebound; the levels of TMAO were higher in individuals who rebounded faster than in individuals who rebounded slower. Mann-Whitney U test was used for statistical analysis. (F) Spearman’s rank correlation between pre-ATI TMAO and post-ATI time to viral rebound. Statistical analyses were performed in R and Prism 7.0 (GraphPad).

We identified a total of 967 lipids, belonging to 21 lipid classes (described in Table S2), in the plasma samples. Using the Cox proportional-hazards model, we found that pre-ATI levels of several of these lipids significantly associated with a faster time to viral rebound (Fig. 1A; lipids with a hazard ratio [HR] of >5 and *P* < 0.01 are labeled). We next examined whether these lipids belong to particular lipidomic pathways or classes. Pathway analysis of all lipids whose pre-ATI levels associated with time to viral rebound with *P* < 0.05 showed that the pathway most associated with viral rebound was glycerophospholipid metabolism (Fig. 1B). The pre-ART levels of three lipid classes were significantly (false-discovery rate [FDR] of <0.05) associated with faster time to viral rebound (Fig. 1C and Table S3): lysophosphatidylcholine (LPC), lysophosphatidylethanolamine (LPE), and lysophospholipid acid (LPA). All three classes belong to the lysophospholipid group, which is a subgroup of the glycerophospholipid family shown in Fig. 1B. Lysophospholipids are small bioactive lipid molecules known to play important roles in regulating several biological functions, including promoting inflammation (8, 14–19). The significant associations between these lysophospholipid classes and faster time to viral rebound were confirmed using two additional, independent analyses: Mantel-Cox survival test, after separating participants into low or high groups based on the median level of each of these lipid classes (Fig. 1D); and Spearman’s rank correlation between the levels of these lipid classes and time to viral rebound (Table S3). These data point, for the first time, to plausible links between phospholipid and lysophospholipid metabolism and HIV rebound post-ART. Intriguingly, similar phospholipids and lysophospholipids were recently shown to reactivate latent (dormant) cancer cells (4). Our exploratory findings, that are consistent with the reported functions of these lysophospholipids, raise the question of whether these lysophospholipids condition the host environment with higher levels of inflammation that might impact viral reactivation, cellular processes, and immunological functions during and/or after ATI.

10.1128/mBio.03444-20.2 TABLE S2 967 lipids identified in this study were assigned to 21 lipid classes. Download Table S2, PDF file, 0.01 MB. Copyright © 2021 Giron et al. 2021 Giron et al. https://creativecommons.org/licenses/by/4.0/ This content is distributed under the terms of the Creative Commons Attribution 4.0 International license.

10.1128/mBio.03444-20.3 TABLE S3 A list of lipid classes whose pre-ATI levels associate with faster time to viral rebound upon ART cessation. Download Table S3, PDF file, 0.1 MB. Copyright © 2021 Giron et al. 2021 Giron et al. https://creativecommons.org/licenses/by/4.0/ This content is distributed under the terms of the Creative Commons Attribution 4.0 International license.

### Levels of plasma trimethylamine-*N*-oxide measured pre-ATI associate with post-ATI time to viral rebound.

The proinflammatory lipid class LPC can be hydrolyzed in the intestine to LPA and choline; choline can be metabolized into trimethylamine, which is converted to trimethylamine-*N*-oxide (TMAO) in the liver (20). TMAO induces several proinflammatory mediators and has been implicated in several inflammation-associated diseases (20–23). Given that LPC and LPA lipids were among the lipids whose pre-ATI levels associated with faster viral rebound upon ART cessation (Fig. 1A to D), we sought to examine whether levels of TMAO associated with post-ATI time to viral rebound. We performed metabolomic analysis, using LC-MS, as described previously (24), on the same pre-ATI plasma samples. Indeed, pre-ATI levels of TMAO were higher in individuals with lower than the median days to viral rebound (fast rebounders) compared to individuals with higher than the median days to rebound (delayed rebounders) (Fig. 1E). Furthermore, pre-ATI TMAO levels correlated negatively with post-ATI time to viral rebound (Fig. 1F). Our observations that the proinflammatory by-products of intestinal LPC metabolism (LPA and TMAO) are also associated with faster HIV rebound demand a greater understanding of the interaction between glycerophospholipid or choline metabolism by intestinal microbiota and viral persistence during ART or rebound post-ART. Such understanding may inform therapeutic approaches targeting the gut microbiota-lipid metabolism interface to reduce inflammation and facilitate the clearance of HIV reservoirs.

### Pre-ATI plasma lysophospholipids associate with post-ATI viral load set point.

**FIG 2:**
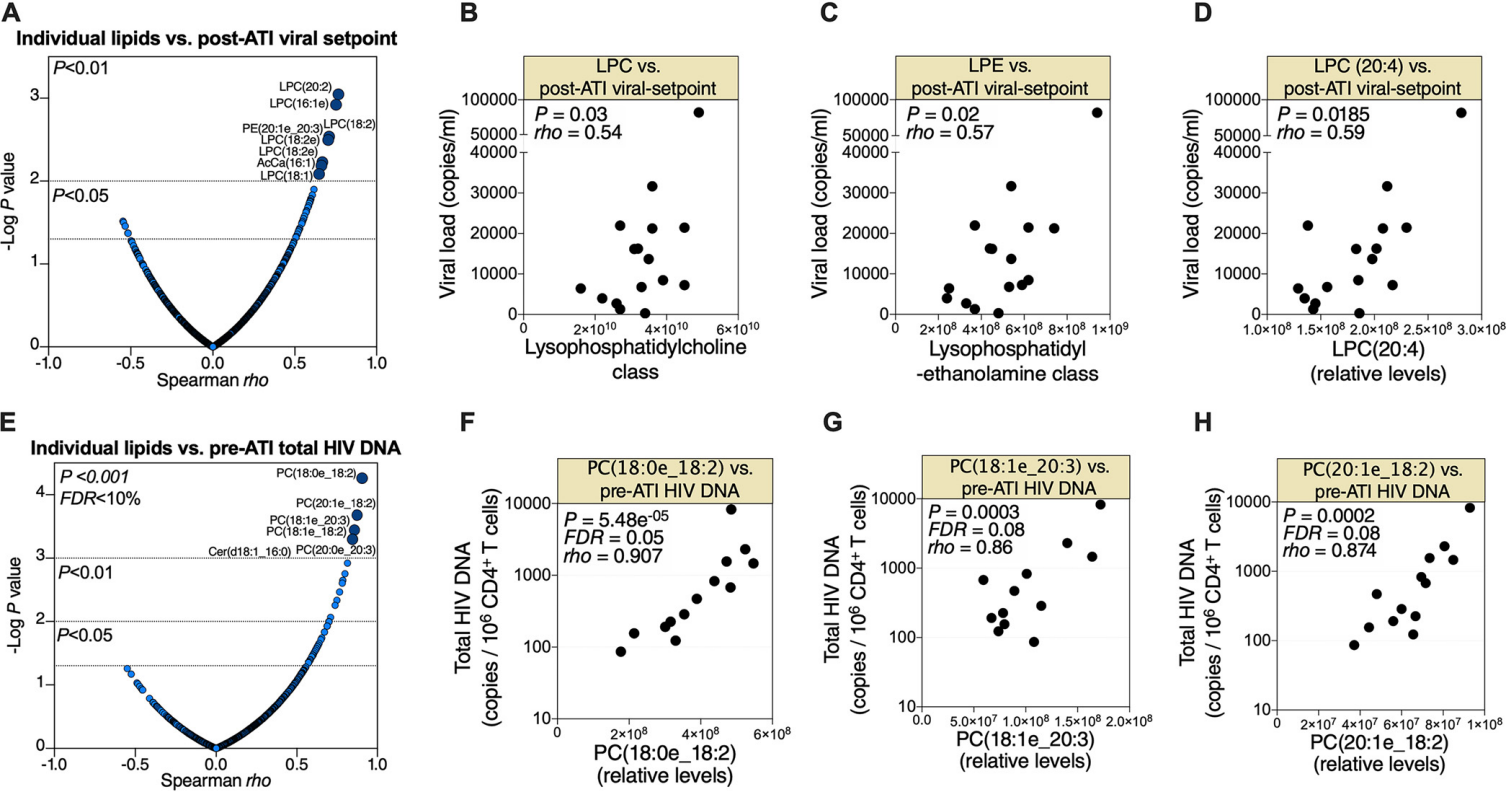
Pre-ATI phospholipid metabolism associates with post-ATI viral load set point and pre-ATI HIV DNA. (A) Spearman’s rank correlations between pre-ATI lipids and post-ATI viral load set point. Lipids with *P* < 0.01 and Spearman rho > 0.5 are shown in dark blue and are labeled. (B to D) Correlations between pre-ATI levels of LPC class (B), LPE class (C), or LPC (20:4) lipid species (D) and post-ATI viral load set point. Each symbol shows the value for one HIV-positive individual. (E) Spearman’s rank correlations between pre-ATI lipids and pre-ATI total HIV DNA measured in peripheral CD4^+^ T cells. Lipids with FDR < 0.1 and Spearman rho > 0.5 are shown in dark blue and are labeled. (F to H) Correlations between pre-ATI levels of several phosphatidylcholine species and pre-ATI levels of HIV DNA in peripheral CD4^+^ T cells. All correlations were evaluated using Spearman’s rank correlation coefficient tests. Statistical analyses were performed in R and Prism 7.0 (GraphPad).

In addition to time to viral rebound, post-ART viral load set point can reflect the body burden of HIV during ART. Therefore, we asked whether pre-ATI lipid profiles are associated with post-ATI viral load set point. Pre-ATI levels of several lipids associated with post-ATI viral load set point with *P* < 0.01 and Spearman rho > 0.5 (Fig. 2A). Furthermore, pre-ATI LPC and LPE class levels correlated with post-ATI viral load set point (Fig. 2B and C, respectively). Finally, the pre-ATI levels of the LPC (24:0) lipid species, which was one of the individual lipids whose pre-ATI level correlated with time to viral rebound (Fig. 1A), also associated with post-ATI viral load set points (Fig. 2D). Notably, levels of LPC (20:4) during HIV infection have been shown to associate with the progression of carotid artery atherosclerosis, even after ART suppression (6). These data indicate that pre-ATI phospholipid metabolism is linked to viral load set point upon ART cessation.

### Pre-ATI phosphatidylcholines associate with pre-ATI HIV DNA in the periphery.

Finally, we examined the links between pre-ATI plasma lipidome and pre-ATI total HIV DNA measured in periphery CD4^+^ T by droplet digital PCR (ddPCR), as described previously (11). Levels of several phosphatidylcholine species (precursors of lysophosphatidylcholine) significantly correlated (FDR <10%) with CD4^+^ T cell-associated HIV DNA (Fig. 2E to H). These data further highlight the potential links between phospholipid metabolism and HIV persistence.

Our exploratory study has limitations, including small sample size and sampling of blood. The sample size did not allow for addressing the confounding effects of age, gender, ethnicity, weight, diet, duration of infection, duration on ART, ART regimen, or comorbidities on lipidomic signatures. Addressing the impact of these confounders and validating our data using larger cohorts should be the subject of future studies. In addition, it will be important, in future studies, to examine the links between phospholipid levels, viral rebound, and established clinical measurements of total cholesterol, high-density lipoprotein (HDL), low-density lipoprotein (LDL), and triglycerides (TG). Finally, analyzing lipids and HIV burden in different tissues, including adipose tissue, and mechanistic studies *in vitro* and in animal models of HIV infection will be needed to examine the precise interplay between phospholipid metabolism and viral persistence. Such studies might identify lipid-based interactions that can be targeted to decrease the size of HIV reservoirs and/or delay viral rebound after stopping ART.

Despite these limitations, our study provides the first proof-of-concept evidence that phospholipid metabolism might be involved in a host milieu that facilitates a faster HIV rebound after ART cessation. The potential interactions between phospholipid/lysophospholipid metabolism and both the establishment and maintenance of HIV latency warrant further investigation.
