# Supplementary material for: Phospholipid Metabolism Is Associated with Time to HIV Rebound upon Treatment Interruption
Source: mBio. 2021 Feb 23;12(1):e03444-20. doi: 10.1128/mBio.03444-20 (PMC8545116; doi:10.1128/mBio.03444-20)
Supplement: TABLE S1 [file mbio.03444-20-st001.pdf]

Supplementary Table 1. Demographic characteristics of the study cohort

Patient ID	Age (years)	Gender	Race	Pre-ATI viral load (copies/ml)	Pre-ATI CD4 count (cells/mm^3)	Pre-ATI CD4%	Post-ATI viral setpoint (copies/ml)	Time to viral rebound (days)
S-8	53	Male	Caucasian	<50	426	23.7	NA	14
S-14	29	Male	Caucasian	<50	782	46	16153	28
S-20	23	Male	African American	<50	708	37.3	3961	28
S-21	40	Male	Caucasian	<50	509	28.3	31674	14
S-25	36	Female	African American	<50	886	40.3	2693	70
S-28	48	Male	Hispanic	<50	478	47.8	274	28
S-29	45	Male	African American	<50	513	15.1	NA	28
S-30	45	Male	Caucasian	<50	602	24.1	NA	14
S-31	45	Male	Caucasian	<50	712	28.5	6775	27
S-32	41	Male	Caucasian	<50	864	37.6	21961	35
S-33	49	Female	African American	<50	901	39.2	NA	15
S-35	51	Male	Caucasian	<50	516	21.5	7260	14
S-36	40	Male	Caucasian	<50	517	39.8	NA	35
S-38	42	Male	Caucasian	<50	688	32.8	8460	21
S-41	54	Male	Caucasian	<50	766	36.5	21246	28
S-42	42	Male	Caucasian	<50	734	36.7	NA	14
S-43	50	Male	Caucasian	<50	690	23.8	13675	28
S-44	44	Male	Caucasian	<50	374	18.7	81949	14
S-49	54	Male	African American	<50	557	42.9	NA	119
S-51	53	Male	Caucasian	<50	434	36.2	1297	14
S-54	43	Male	Caucasian	<50	832	34.7	21456	14
S-55	38	Male	African American	<50	615	32.4	NA	42
S-60	51	Male	Caucasian	<50	669	39.4	16249	21
S-62	48	Male	African American	<50	720	42.4	6423	42
