# Supplementary material for: Phospholipid Metabolism Is Associated with Time to HIV Rebound upon Treatment Interruption
Source: mBio. 2021 Feb 23;12(1):e03444-20. doi: 10.1128/mBio.03444-20 (PMC8545116; doi:10.1128/mBio.03444-20)
Supplement: TABLE S2 [file mbio.03444-20-st002.pdf]

Supplementary Table 2. Nine hundred sixty-seven lipids identified in this study were assigned to 21 lipid classes.

Group	Abbreviation	Class	Number of lipids
Phospholipids	LPA	Lysophosphatidic acid	2
	PA	Phosphatidic acid	8
	LPC	Lysophosphatidylcholine	91
	PC	Phosphatidylcholine	173
	LPE	Lysophosphatidylethanolamine	18
	PE	Phosphatidylethanolamine	117
	PG	Phosphatidylglycerol	10
	LPI	Lysophosphatidylinositol	2
	PI	Phosphatidylinositol	46
	PS	Phosphatidylserine	2
Neutral lipids	ChE	Cholesterol ester	10
	DG/DAG	Diglyceride	19
	TG/TAG	Triglyceride	201
Sphingolipids	Cer	Ceramide	58
	Hex1Cer	Simple Glc series (Ceramide with 1 hexose)	3
	Hex2Cer	Simple Glc series (Ceramide with 2 hexose)	5
	Hex3Cer	Simple Glc series (Ceramide with 3 hexose)	4
	LSM	Lysosphingomyelin	1
	SM	Sphingomyelin	159
Other lipids	AcCa	Acyl carnitine	36
	Co	Coenzyme	2
