# Supplementary material for: Phospholipid Metabolism Is Associated with Time to HIV Rebound upon Treatment Interruption
Source: mBio. 2021 Feb 23;12(1):e03444-20. doi: 10.1128/mBio.03444-20 (PMC8545116; doi:10.1128/mBio.03444-20)
Supplement: TABLE S3 [file mbio.03444-20-st003.pdf]

Supplementary Table 3. A list of lipid classes whose pre-ATI levels associate with faster time-to-viral-rebound upon ART cessation.

		Proportional Cox Hazard Model					Mantel Cox Test		Spearman's correlation		
**Lipid class**	**Group**	**HazardRatio (HR)**	**HR 95% lower confidence limit**	**HR 95% higher confidence limit**	***P* value**	***FDR***	***P* value**	***FDR***	***P* value**	***FDR***	***rho***
**Lysophosphatidylethanolamine**	Phospholipids	4.255	1.586	11.419	0.004	0.028	0.006	0.064	0.002	0.023	-0.595
**Lysophosphatidylcholine**	Phospholipids	6.340	1.992	20.181	0.002	0.019	0.023	0.160	0.002	0.023	-0.608
**Lysophosphatidic acid**	Phospholipids	6.970	2.063	23.548	0.002	0.019	0.040	0.212	0.006	0.036	-0.548
**Phosphatidylinositol**	Phospholipids	2.808	1.082	7.290	0.034	0.147	ns		0.014	0.058	-0.496
**Ceramide**	Sphingolipids	3.303	1.018	10.714	0.047	0.147	0.002	0.042	0.007	0.036	-0.536
